# Editorial: Neuro-inspired computing for next-gen AI: Computing model, architectures and learning algorithms

**DOI:** 10.3389/fnins.2022.974627

**Published:** 2022-07-25

**Authors:** Angeliki Pantazi, Bipin Rajendran, Osvaldo Simeone, Emre Neftci

**Affiliations:** ^1^IBM Research - Zurich, Switzerland; ^2^Department of Engineering, King's College London, London, United Kingdom; ^3^Department of Cognitive Sciences, University of California, Irvine, Irvine, CA, United States

**Keywords:** neuromorphic computing, Spiking Neural Networks, neuronal model, learning algorithms, neuromorphic hardware

## Introduction

Today's advances in Artificial Intelligence (AI) have been primarily driven by deep learning and have led to astounding progress in several tasks such as image classification, multiple object detection, language translation, speech recognition and even in the ability to play strategic games. However, the AI systems of today have several limitations. Specifically, the hardware infrastructure is limited to high-power and large-scale processing systems that are based on the von Neumann computing paradigm. Moreover, there is a growing demand for applications with cognitive functionality that will be able to operate in real time and in an autonomous manner in the field. The limitations of contemporary AI systems are in stark contrast to the capabilities of the brain which can learn and adapt very quickly consuming just about 20 W of power.

Neuromorphic computing, inspired by neuroscience, is a promising path toward the next-generation AI systems. The research focuses on different levels of the design stack, i.e., the computing model, the architecture and the learning algorithms. The computing model is based on Spiking Neural Networks (SNNs), which possess more biologically realistic neuronal dynamics as compared to those of Artificial Neural Networks (ANNs). At the architectural level, SNNs implement in-memory computing, which is well suited for efficient SNN hardware realizations. At the algorithmic level, neuro-inspired learning paradigms are based on the insight that the brain continuously processes incoming information and is able to adapt to changing conditions. Thus, online learning, learning-to-learn, and unsupervised learning provide the main conceptual platforms for the design of low-power, accurate and reliable neuromorphic computing systems.

This Research Topic provides an overview of the recent advances on computing models, architecture, and learning algorithms for neuromorphic computing. In the rest of this Editorial, we provide a brief description of the accepted papers contributing to each of these areas.

## Computing model

State-of-the-art deep learning is based on ANNs that only take inspiration from biology to a very limited extent—primarily in terms of the ANNs' networked structure. This has several drawbacks, especially in terms of power consumption and energy efficiency. More biologically realistic neural models have been considered as promising contenders for the next generation of neural networks. In this Research Topic, Dellaferrera et al. present a biologically-inspired computational model for blind source decomposition based on a two-compartment somatodendritic neuron and synaptic connections trained by Hebbian-like learning. Their results demonstrate blind source separation on a sequence of mixtures of acoustic stimuli, suggesting that the proposed neuronal model can capture characteristics of the brain's segregation capability. Delacour and Todri-Sanial present an emerging neuromorphic architecture in which neurons are represented with oscillators, and the information is encoded in the oscillator's phase relations. They present an oscillatory neural network (ONN) using relaxation oscillators based on VO_2_ material. They demonstrate that an ONN consisting of 60 fully-connected oscillator neurons can implement a Hopfield Neural Network that performs pattern recognition.

## Architectures

The network structure in biological systems provides energy efficiency and low latency, while combining memory and computation. In recent years, ANN-to-SNN conversion techniques have enabled the design of SNNs, starting from well-known ANN architectures, that offer lower computation cost compared to their non-spiking counterparts. Moreover, the concept of in-memory computing, which aims at co-locating the memory and processing units, has recently demonstrated that substantial acceleration may be achieved for ANNs. In this Research Topic, Wu et al. propose a framework for developing an energy-efficient SNN using a novel explicit current control (ECC) method that converts a CNN to an SNN. The key contribution of this framework is that, during the conversion, multiple objectives are considered including accuracy, latency, and energy efficiency. Zou et al. present a hardware-friendly algorithm that converts a quantized ANN to an SNN by minimizing the spike approximation errors that are typically emerging in ANN-to-SNN conversion. Furthermore, they develop strategies for mapping the designed CNN to crossbar-based neuromorphic hardware.

Yan et al. propose a sparsity-driven SNN learning algorithm (BPSR) that incorporates spiking regularization to minimize the neuronal spiking rate. To further mitigate the redundancy of the network structure, they suggest a rewiring mechanism with synaptic regularization. The proposed BPSR scheme improves the spiking and synaptic sparsity while achieving comparable accuracy with related works. Finally, Datta et al. propose a deep SNN for 3D image recognition using algorithmic and hardware co-design approaches, namely quantization-aware backpropagation and processing-in-memory (PIM) architecture. Their results yield low latency (5 time steps) and low bit width (6-bit weights). The adoption of the PIM architecture in the first layer further improves the average energy, delay, and energy-delay-product.

## Learning algorithms

The brain is equipped with impressive learning capabilities, enabling animals to dynamically adapt to the surrounding world. Hebbian learning and Spike-Timing Dependent Plasticity (STDP) are commonly employed learning rules in neuro-inspired models. The convergence properties and computational characteristics remain largely unknown. In this Research Topic, Chakraborty and Mukhopadhyay study the generalizability properties of SNNs equipped with STDP. They achieve this goal by analyzing the dimensionality of the space spanned by the learning process, and propose a method to optimize hyperparameters to improve the network generalization properties.

Neuro-inspired computing has recently taken inspiration from machine learning to implement online learning rules based on gradient descent. These generally transferred the basic modules of deep learning, but lag behind in other components essential to deep learning. One of these components is batch normalization, which is now ubiquitous in deep learning to improve convergence speed and accuracy. In this Research Topic, Kim and Panda showed how batch normalization can be adapted to SNNs, thereby enabling significant acceleration in SNN training.

Compared to gradient descent, STDP has the advantage that it does not require external supervision, and can therefore operate locally in neuromorphic hardware. However, gradient descent generally performs better if such signals exists. In their proposed model, Krithivasan et al. selectively adjust the learning rules employed by the layer during training to exploit the best of STDP and SGD. In an associative learning framework, Mo et al. use external supervision to improve the performance of STDP, and demonstrate successful STDP learning in common labeled machine learning datasets.

## Author contributions

All authors listed have made a substantial, direct, and intellectual contribution to the work and approved it for publication.

## Conflict of interest

AP was employed by IBM Research. The remaining authors declare that the research was conducted in the absence of any commercial or financial relationships that could be construed as a potential conflict of interest. BR's research is partly funded by Intel, Cisco, European Space Agency, and Semiconductor Research Corporation outside of this work. The funder was not involved in the study design, collection, analysis, interpretation of data, the writing of this article or the decision to submit it for publication.

## Publisher's note

All claims expressed in this article are solely those of the authors and do not necessarily represent those of their affiliated organizations, or those of the publisher, the editors and the reviewers. Any product that may be evaluated in this article, or claim that may be made by its manufacturer, is not guaranteed or endorsed by the publisher.

